# Creating a better learning environment: a qualitative study uncovering the experiences of Master Adaptive Learners in residency

**DOI:** 10.1186/s12909-022-03200-5

**Published:** 2022-03-04

**Authors:** Linda Regan, Laura R. Hopson, Michael A. Gisondi, Jeremy Branzetti

**Affiliations:** 1 Department of Emergency Medicine, Johns Hopkins University School of Medicine, 1830 E. Monument Street, Suite 6-100, Baltimore, MD 21093 USA; 2 Department of Emergency Medicine, University of Michigan Medical School, Ann Arbor, MI USA; 3 Department of Emergency Medicine, Stanford University School of Medicine, Stanford, CA USA; 4 Department of Emergency Medicine, New York University School of Medicine, New York City, NY USA

## Abstract

**Background:**

Adaptive expertise is an important physician skill, and the Master Adaptive Learner (MAL) conceptual model describes learner skills and behaviors integral to the acquisition of adaptive expertise. The learning environment is postulated to significantly impact how MALs learn, but it is unclear how these successful learners experience and interact with it. This study sought to understand the authentic experience of MALs within the learning environment and translate those experiences into practical recommendations to improve the learning environment for all trainees.

**Methods:**

Following a constructivist paradigm, we conducted a thematic analysis of transcripts from focus groups composed of MALs to identify commonalities in experiences and practices of successful postgraduate trainees in the learning environment. Saturation was achieved after seven focus groups, consisting of thirty-eight participants representing fourteen specialties from four institutions. Researchers coded transcripts using constant comparison analysis, which served as the foundation for our thematic analysis.

**Results:**

We identified eight themes and situated them within a 4-component model of the learning environment. Four themes were identified within the personal component: (1) patients drive learning; (2) learning has no endpoint; (3) management of emotions is crucial for learning; (4) successful learning requires a structured approach. Two themes were identified in the social component: (5) positive social relationships are leveraged to maximize learning; (6) teaching facilitates personal learning. Two themes were identified in the organizational component: (7) transitions challenge learners to adapt; (8) the learning environment dictates goal setting strategy. No major themes were identified in the physical/virtual component, although participants frequently used technology when learning.

**Conclusions:**

Master Adaptive Learners experience similar facilitators of, and barriers to, success in the learning environment. Overall, our data show that acquisition of many successful strategies and skills that support learning are relegated to the hidden curriculum of residency training. Educators could support a more effective learning environment for all trainees by: *(1) highlighting patients as the focal point of learning, (2) building a professional ‘learner’ identity, (3) teaching learning skills, and (4) creating opportunities for collaborative learning.*

**Supplementary Information:**

The online version contains supplementary material available at 10.1186/s12909-022-03200-5.

## Background

Adaptive expertise is among the most important cognitive skills necessary for successful independent practice in medicine [1–3]. While routine experts can apply foundational knowledge to effectively solve common clinical problems, adaptive experts can *also* transfer their existing knowledge to solve uncommon or new problems [4]. Without developing adaptive expertise, learners may struggle with rapidly changing care environments and the ever-expanding body of knowledge in medicine. Those who do develop adaptive expertise are primed to be effective lifelong learners, and therefore may be best prepared for dynamic, longitudinal medical careers.

Drawing on multiple theories of learning and self-regulation, the Master Adaptive Learner (MAL) conceptual model was proposed to describe the development of adaptive expertise [5]. The authors describe cognitive skills, internal characteristics (curiosity, motivation, mindset, resilience) and external factors (coaching, learning environment) believed to enable a MAL to thrive [6]. These internal characteristics are intuitively valuable, and the potential benefits of academic coaching are well-described in the medical education literature [7, 8]. In contrast, the impact of the learning environment (LE) is more complex and nuanced. Given certain circumstances, the LE can be either highly beneficial to learning, or detrimental and destructive [6, 9]. In a 2018 review, Gruppen et. al. conceptualized the LE as a “complex psycho-social-physical construct co-created by individuals, groups, and organizations in a particular setting, and shaped by contextual climate and culture,” with four overlapping core components [9]. The *personal component* focuses on the psychological and experiential aspects that lead to the growth and development of the learner. The *social component* refers to interpersonal relationships in the LE between learners and peers, supervisors, staff, and patients. The *organizational component* encompasses any structure, guidance, and support for learning within the LE. Lastly, the *physical and virtual component* is the actual material space of the LE and the information technology resources that make up the virtual environment.

The characteristics that help a MAL thrive as a learner may also help them adapt to a wider range of LEs, particularly adverse ones [6, 9]. How they do so is less clear, as empirical evidence of MAL strategies in the LE is lacking. In this study, we aimed to better understand how Master Adaptive Learners experience and interact with the LE during residency training. Capitalizing on the input of these high-performing learners, we translate their experiences into practical recommendations to improve the LE for all trainees.

## Methods

We conducted focus groups exploring the following two questions: (1) how MALs plan their learning during GME training, and more generally, (2) how they approach their practice of learning within the learning environment of GME training. Constant comparison analysis yielded 205 codes, a subset of 47 were applicable to the first, more narrowly focused study aim about the planning of learning [10]. In this study, we used a constructivist paradigm [11] to conduct a thematic analysis of the entirety of our coded data to explore the second question.

### Study participants

We used purposeful sampling to recruit a cohort of resident trainees from major clinical specialties for focus group interviews. We provided the MAL framework to residency directors at the four authors’ institutions and asked them to refer residents whom they felt demonstrated key attributes of MALs. Those referred residents were then approached via email to participate in a study of “how you learn most effectively.” Saturation of themes [12] occurred after the seventh focus group, yielding a sample consisting of 38 residents across 14 specialties (Table 1).

**Table 1:** Demographic characteristics of focus group participants

**Gender**	**Number (%)**	
Male	24 (63%)	
Female	14 (37%)	
**Specialty Type**	**Number (%)**	**Specialties Represented**
Medical	13 (34.2%)	Internal Medicine, Medicine-Pediatrics, Pediatrics, Neurology, Physical Medicine & Rehabilitation
Surgical	6 (15.8%)	Obstetrics and Gynecology, Orthopedic Surgery, Otolaryngology, Plastic Surgery, Urology
Hospital-based	19 (50.0%)	Anesthesiology, Diagnostic Radiology, Emergency Medicine, Pathology

### Study sites

All four sites are university-based medical centers sponsoring numerous training programs. They are located in the Northeast, Mid-Atlantic, Mid-West, and West Coast regions of the United States. IRB approval was obtained at all sites.

### Focus group guides

Building on the description of the MAL model [5], our interview guide included open-ended question prompts about how participants planned their learning and broader questions about their overall experience of learning. The lead investigator designed the interview guide and the entire study team reviewed, revised, and finalized it. A convenience sample of educators reviewed the guide for content and relevance, with minor edits for clarity made after piloting the guide with a group of trainees in a medical education development program at one of the sites. Pilot data was not included in the analysis. Use of the guide was standardized across all groups to maximize consistency in data collection. The guide is available as a supplemental appendix.

### Data collection

Five moderators led seven audio-recorded focus groups across four sites. All focus groups contained a mix of residents from across specialties. Sessions ranged from 65–94 min, with an average of 5 participants per group (range 2–9). To encourage open discussion, participants used pseudonyms and we encouraged the use of moderators not involved in the supervision of participants. We downloaded audio recordings to a secure server for transfer to a third party for transcription. Member checks were used once final analysis was done [13].

### Data analysis

Transcripts were coded independently, in a blinded fashion, by two study investigators using Dedoose (Version 8.1.8, 2018, Los Angeles, CA: SocioCultural Research Consultants, LLC). After initial review and coding, the primary investigator unblinded assigned codes for group review, with a third investigator adjudicated coding discrepancies. Iterative analysis occurred using a master codebook until saturation was reached. Thematic analysis of the data yielded eight themes related to how participants approached learning in the LE, which we situated within a 4-component conceptual framework of the LE [9].

### Reflexivity

We acknowledged the potential impact of our past experiences during data analysis in this constructivist paradigm. All four authors are emergency physicians and past/current residency program directors. We reflected on this potential influence at the start of the study and throughout data analysis to minimize bias.

## Results

Our thematic analysis found that this representative group of MALs experienced and navigated the LE very similarly. Our key findings are presented through the lens of the four-component conceptual framework proposed by Gruppen et. al. [9] focused on the LE: (1) personal component, (2) social component, (3) organizational component, and (4) the physical and virtual component.

### Personal component

The personal component refers to the learner’s interactions in the LE that impact their perceptions, decisions around their learning processes, and overall growth and development. This component was manifested through four themes that collectively situate the patient at the center of a continuous, unending process of learning that is actively grown and refined by an emotionally grounded MAL.

1. ***Patients drive learning***

Participants repeatedly identified patients as the centerpiece of training, serving as the primary source of and purpose for learning during their post-graduate training. This focus on patients distinguishes learning in residency from previous experiences.

The most significant motivator for developing expertise was a fundamental sense of responsibility to patients. This internal motivation dictated what, when, and how they learned.

‘[P]atient care is the fundamental mission and goal of what we are doing. And the process of learning is what happens as a result of that. And I think . . . if you put the patient at the center of what you were doing at all times, everything else will fall into place.’

‘It totally has to come from within. . . That’s what the goal should be for residency education - it should really be to remind people that you’re responsible for the person’s medical care and it should be an internal set of motivations rather than a test you just have to study for.’

Specific patient care experiences provided memorable clinical examples that led to more effective retrieval of foundational knowledge.

‘Once you have the face, you remember – when someone mentions a disease, you automatically remember this whole clinical experience from first seeing the patient to whatever the outcome was. It . . . almost comes like in the flash of a second.’

2. ***Learning has no endpoint***

Participants described learning medicine as an intrinsically rewarding, career-long journey. They recognized that this learning extends beyond any single task or training program; it is an unending process of continuous self-improvement. MALs viewed learning challenges as growth opportunities, highlighting that true learning did not come in the form of a quick fix (e.g. cramming for a test).

‘One of my first attendings in medical school told me, be kind to yourself, and understand that medicine is sort of a journey. . . If you still aren't learning things when you’re 70, then you’re doing something wrong. . . And I think because of that, I’ve been able to accept that I’m in for a long ride and not feel rushed to learn everything rapidly, and if I can just learn a couple of new things a day, then I’m on the right track.’

‘ If you identify this idea of, ‘you’re a learner now,’ it implies the idea that you are *not* a learner in the future. And that is very much not the case. The best physicians – we all know – are the people who are learning constantly, and the people who love to learn.’

3. ***Management of emotions is crucial for learning***

The dominant motivational factor discussed by participants was their emotional response to events during training. Negative emotions were both more commonly cited and more impactful. Emotions such as inadequacy served as vivid reminders of experiences or mistakes that participants wanted to avoid repeating.

‘I do think that each time I’ve had those experiences, it has changed dramatically how I approach the next time. I think that’s something where it just scares the crap out of you so much, ‘I’m never going to make this mistake again.’

Participants also described the need to manage emotions, such as shame and disinterest towards learning (i.e., the *absence* of emotional engagement), to successfully move forward in their learning.

‘I think the other thing I learned about myself, and this is incredibly helpful for me, was that stress makes everyone feel really dumb. Anxiety and stress, they make it very hard to think for me. And everyone deals with this differently, right? But for me in particular, something that I had to understand about myself was that it’s not that I’d become dumber. It’s that I am tired and stressed, and that combination is really affecting my ability not only to process information, but also to learn and retain.’

‘Do you remember where you were when you first met that person or when you were engaged? There’s a lot of emotion with that, right? And I think that helps memory versus if you say, okay, where did you first get your oil changed? I mean, no one knows that. Who cares? So, if you can make it meaningful it becomes more memorable.’

4. ***Successful learning requires a structured approach***

Participants described challenges to develop effective learning habits in the new, unstructured LEs of residency: ‘You feel as if you’re on a boat in choppy water trying to build a house.’

Most participants did not receive guidance from their residency programs on how to create learning plans or select learning resources. All participants described the need to develop their own effective approach to learning, refined over time through accrued personal experience. This centered upon the use of mental schemata to organize foundational content necessary for clinical decision-making.

‘You come up with an approach to each situation you can be in and then develop fallbacks. So, I just started right away when I was an intern making a playbook: [H]ow am I going to do this? How am I going to go about it?. . . And then you can bring that and test it out at work.’

Development of these schemata is effortful, iterative, and internally driven, facilitated by patient care experiences.

‘I have . . . branches that I think of. What I’ve found over the course of residency is that things grow. And that space where you have the chunk or the hook, you keep adding details into that – fine details. And sometimes you have to go back and re-imagine the whole thing. . . But it keeps expanding. I found [it’s] a really helpful process to realize learning is iterative.’

### Social component

The social component encompasses the innumerable interpersonal relationships and experiences that shape the learner’s engagement with the LE. Our participants leveraged both vertical interactions with supervising attendings/near peers, and horizontal interactions with peers, to maximize their learning. Teaching was noted to be an especially beneficial interaction for personal learning.

5. ***Positive social relationships are leveraged to maximize learning***

Participants identified vertical and horizontal social interactions that contributed significantly to their learning in the form of *empowerment,* identification of *trusted sources*, and *communities of practice*.

#### Empowerment

Entrustment by a senior team member empowered the participants, who were profoundly aware of the responsibility that came with decision-making of autonomous patient care.

‘[E]mpowerment to make that decision in a supportive environment where there’s supervision is the key to growing and learning . . . as a clinician.’

‘I have seen other interns or residents not get as much out of their experiences. . . As soon as the question comes up – they go to their senior or they go somewhere else, and they don’t necessarily take ownership. . . I think if you do take ownership . . . that’s when the learning aspect happens*.’*

Conversely, learning opportunities were lost when participants were excluded from clinical decision making and relegated to passive, peripheral observers of patient care.

‘When did this get added on? When did this change? They [say], “Oh, you were off doing something else.” Stuff just happens without me knowing it . . . when you don’t have full ownership of a patient.’

#### Trusted sources

Some subjects identified specific faculty members and near peers as trusted sources for information to assist them across multiple stages of the MAL cycle: assistance with the identification of their gaps (Planning Phase); soliciting expert opinions on recent articles (Learning Phase); and soliciting feedback on the application of new knowledge (Assessing and Adjusting Phases). The content expertise and opinions of these learner-selected sources were critically important.

‘Most of my learning is coming from people above me ... I have particular attendings that I have a lot of confidence in, and I trust their clinical expertise.’

Finally, participants valued horizontal learning across disciplines, viewing peer consultants as trusted experts.

‘I also think that we are very lucky to be at a place that has residencies in all specialties . . . and it’s very easy to get feedback from them, sort of lateral feedback. ... We often bounce cases off each other: How would you deal with this? What’s your workup of this?’

#### Communities of practice

The majority of participants highlighted the value of horizontal peer learning. These interactions occurred organically during clinical care and in resident-led teaching sessions outside of the clinical space. Two representative examples of peer-learning included: (1) a group of senior residents who voluntarily gathered before rounds to collaboratively read and plan daily teaching for their teams and (2) diagnostic image review sessions in which trainees submitted their errors for resident-only review and feedback.

‘We ask residents to submit [imaging] cases that they missed. . . We ask everyone in the residency to take a look at the same things that the person missed, and they try to find it. And sometimes they do and sometimes they don’t. And that’s kind of reassuring, also, to know that other people could also make the same mistakes – not that we should, but we learn from those mistakes, and we try to change because of it.’

6. ***Teaching facilitates personal learning***

Teaching was an effective method of learning for MALs, serving as: (1) a means of gap identification; (2) a motivator for their learning; (3) a process for expanding and reinforcing their foundational knowledge; (4) a marker of *adequacy* [10] in learning; (5) a means to identify the underlying conceptual knowledge being learned; and, (6) a demonstration of the ability to effectively communicate about a topic. Most intentionally used teaching as a fundamental learning strategy. Those who received training to be better teachers described a dual outcome of the training: simultaneously improving their skills as teachers and their own study habits as learners.

‘I don’t know that I’ve learned how to learn; but in learning how to teach, we’ve been taught about adult learner theory ... and the different ways that people learn, which you can reflect back on yourself.’

### Organizational component

The organizational structure of the LE affects nearly all aspects of the training experience. Though much of this structure ostensibly exists to support learning (i.e., accreditation requirements, program policies, varied clinical rotations, etc.), these manifestations were often paradoxically experienced as impediments to learning. Identified barriers included the struggle to transition between LEs (e.g., between medical school and residency, and between clinical rotations) and the need to adapt learning goals to the (un)predictability of LEs.

7. ***Transitions challenge learners to adapt***

The transition to graduate medical education challenged participants to navigate a LE that was far less structured than medical school. This transition prompted an evolution in the focus of their learning from examinations (e.g., ‘book knowledge’) to patients (e.g., clinically meaningful knowledge application).

‘I find myself not worrying about tests at all… [I] focus on what is going to make me the best clinician. I think that my mindset has totally changed.’

‘I’ve realized that clinical information and test information are two completely different things. Because in real life, your clinical information is not that straightforward. It’s not that black and white.’

Compounding the challenge of this transition, participants experienced significant uncertainty in assessing their overall progress and performance as a trainee. They worried about how to recognize their own competence in the clinical environment without the convenience of defined metrics (e.g. tests).

‘I think that it’s really, really challenging to measure how well a resident is doing. I don’t think that our tests or even our formal feedback process is that great at capturing a good resident versus a bad resident. . . in med school, you get a number. [Now] it’s such a complex thing that it’s almost impossible for me to see how it’s measured.’

The transition between clinical rotations also challenged our participants, as they described how they had to learn how to operate in each new LE *before* they could focus on learning medical content. Each change in rotation produced a predictably burdensome cognitive load that included new patient care processes, interpersonal relationships, electronic health records, and administrative burdens.

‘Understanding the flow of what’s going on [comes] first . . administratively, like where things are located, what things I need for what. And then the following week is just going to be getting used to what it is that we actually do in this particular rotation. And I think second [and] third [week] is more of learning and . . . the last week is really enjoying it and just getting more experience.’

8. ***The learning environment dictates goal setting strategy***

The LE directly influenced the utility of learning goals for our participants. On specialty rotations, or prior to participating in a planned surgery, learners embraced prospective goal setting.

‘I do a lot of subspecialty rotations, and this is . . . my one shot to get [an] experience. . . I’ve got to hit that by the end of this [rotation], because there’s a possibility [that] if I don’t, I’ll never get that knowledge.’

However, setting goals was felt to be counterproductive in clinical settings with undifferentiated patients, such as the emergency department. Instead, learning was opportunistic and based on patient care experiences, the curiosity of the participant, and self-identified knowledge gaps.

‘One of my least favorite questions, when I show up to a shift [in the ER], is “what do you want to learn today?”. . .There’s going to be certain patients that show up, and I’m going to learn about whatever they have.’

‘I don’t really set goals for my learning. . . I’ll ask if I can improve whatever I was doing. Or what’s the next question? So that leads to another cycle of getting curious about something, asking myself the right questions and learning about it. So, it’s not a hard learning objective or goal, because I don’t really think that makes sense ...it’s not really how you build long-term effective knowledge.’

### Physical and virtual component

Our participants did not discuss the physical layout of the LE, but frequently referenced the virtual component in the form of specific apps, devices, data aggregation websites, and learning management systems to effectively catalogue knowledge gaps and organize their evolving illness scripts and mental schema.

‘I put [content] into an online database. I use Evernote^Ⓡ^ [Redwood City, California, USA]. . . I can reference it in any kind of scenario on my phone or on a laptop or a desktop.’

## Discussion

The MAL conceptual framework postulates that the LE significantly influences MAL development [6], and our findings identify a variety of factors that contribute to their learning success during residency training. Furthermore, these findings offer a roadmap to optimize workplace learning for all by capitalizing on the strategies used by these successful MALs.

Though our themes are organized under the various components of the LE, they are unified by a common reality for learners in residency: acquisition of successful learning skills and strategies is not taught, and thus relegated to an implicit “hidden” curriculum. This unplanned curriculum fills the gap between the “designed and experienced curricula” [14], and often leads learners to internalize suboptimal behaviors or skills. This, in turn, can yield downstream consequences such as negative impacts on career selection, achievement of clinical competency, and development of professional values [15, 16]. One proposed method to counteract these negative impacts and improve the LE culture is to align it with its desired core values by making the “hidden visible and the implicit explicit” [17].

MALs exhibit traits, behaviors, and preferences that allow them to navigate the hidden curriculum of the LE. We translate their successes at this endeavor into recommendations for program leaders to improve the LE for all and recommend four broad directives that should be made visible to all learners. Practical implementation tips are provided. (Table 2).

**Table 2:** Recommendations for educators

Recommendation	Rationale	Practical Suggestions for Implementation
*Highlight patients as the focal point of learning*	Patients help to facilitate deeper knowledge by stimulating curiosity, serving as motivators to learn, and generating emotions	•Use the learner’s own patient experiences to guide learning and coaching sessions • Normalize the emotional experiences of training through collaborative storytelling events [18, 19] •Teach cognitive reappraisal[20] to harness the power of emotions in learning
*Build a Professional ‘Learner’ Identity*	Internalization of a ‘learner’ identity primes trainees to view learning as intimately bound to their role as physicians	•Introduce, and normalize, concepts like growth mindset, productive struggle, and productive failure [21, 22] •Build a learning culture: Encourage bi-directional learning with both residents and faculty being inquisitive and receptive to questions about clinical decision-making •Use “what if…” questions to build hypothetical variability to clinical encounters to model adaptability as a normal part of physician experience [23]
*Teach Learning Skills*	Trainees do not have adequate preparation to learn in the unstructured environment of residency training	**Learning Skills** •Teach evidence-based learning skills [24] (e.g., interleaving, spaced repetition) and previously identified MAL skills [6] (e.g. weighted curation, triage, and adequacy) •Emphasize conceptual knowledge by focusing learners on the ‘why’ over the ‘what’ •Encourage organizational practices (e.g., apps) to help track knowledge gaps, illness scripts, and mental schemata **Transitions** •Communicate the struggles and skills of transitions with learners during orientation •Cultivate learner self-reflection/self-awareness, organization, and social connections [25] •Teach adaptable goal setting (i.e. prospective for predictable LEs, and opportunistic for unpredictable LEs.) **Teaching** •Build a culture of teaching (e.g. maximize teaching opportunities, celebrate excellent resident/faculty teachers) •Develop evidence-based teaching skills
*Create opportunities for collaborative learning*	The learning environment contains a deep reservoir of vertical and horizontal social learning opportunities	•Prompt learners to identify trusted sources during formal evaluation or coaching sessions •Provide trainees with encouragement, time, and space to generate peer learning collaborations •Maximize learner autonomy commensurate with ability [26]

### Highlight patients as the focal point of learning

Similar to other studies [27–30], we found that patients motivate our learners, improve their foundational knowledge, and promote the deep conceptual understanding associated with adaptive transfer [31]. Advocating for patient-centered learning is not new [32, 33], but the modern clinical LE remains riddled with cumbersome electronic health records (EHRs), process metrics, and administrative burdens that pull learners from the bedside [17, 34, 35]. Structuring education around direct patient care sparks learner curiosity, promotes a sense of ownership that leads to professional responsibility, and stimulates emotions that facilitate deeper learning of clinical content [36, 37].

An important manifestation of this patient-focused learning was the motivating effect of emotions experienced during learning. The most common motivation was the desire to avoid re-experiencing a negative emotion, such as disappointment or shame. What stood out about our participants was their ability to “cognitively reappraise” their emotions [20], repurposing them into powerful motivators to remedy their underlying performance gaps. This emotional undercurrent is another example of the hidden curriculum of learning, where suppression of emotion or dysfunctional reappraisal can occur with deleterious effects on the learner [38].

By truly centering learning around patients, we may finally address the pernicious resident belief that patient care tasks and learning are competing priorities [28]. We recommend that experience with patients serve as the foundation for both learning and learner coaching, and that factors that encourage ownership be considered (e.g. maximizing learner autonomy with appropriate supervision and commensurate with ability [39], increasing rotation length [26, 40]). In addition, collaborative events allowing learners to talk about emotionally impactful patient encounters [18, 19] may normalize the influence of emotion, and encourage its productive use, in learning.

### Build a professional ‘Learner’ identity

Professional identity is defined by Cruess et. al [41]. as “a representation of self, achieved in stages over time during which the characteristics, values, and norms of the medical profession are internalized, resulting in an individual thinking, acting, and feeling like a physician.” Professional identity formation (PIF) is postulated to occur via a complex process of socialization in which both conscious and unconscious processes build a new professional and personal identity, relying heavily on the interactions with role models, mentors, clinical and non-clinical experiences [42]. As with our findings, the critical experience of PIF is heavily reliant on the unspoken learning values and priorities found in the hidden curriculum.

Given the need for future physicians to continuously adapt to rapid changes, it is essential that program leaders explicitly include “lifelong learner” within the professional identity of a successful physician. One important component of this identity to model is that struggle can be productive to learning. This “productive struggle” [21, 22] is a core developmental tenet of adaptive expertise [31, 43, 44] and is consistent with inclusion of growth mindset as an essential trait of MALs [6]. Just as importantly, educators need to create an environment of inquiry that allows for learners to understand that knowledge will need to be adapted for future use. Educators can use “what if…” questions that safely build hypothetical variability and escalating complexity into actual patient cases to help understand, accept, and ultimately internalize productive struggle [23] and adaptability as a part of normal everyday learning.

### Teach learning skills

Consistent with previous reports, our participants worked to adapt medical school learning habits to the less structured LE of residency with minimal program support [10, 25, 45]. Given the persistence of this problem, we advocate that program leaders explicitly include the science of learning alongside the traditional clinical content of residency. A number of learning skills can be role modeled through thoughtful curricular design (e.g. interleaving, retrieval practice [24]), but we highlight two unique ones not often considered to be learning skills: *transitions* and *teaching*.

We recommend shifting the locus of control for transitions by reframing them as a skill that can be cultivated *within* learners and not an event that happens *to* them. In response to the detrimental effect of transitions on learners, Chang et. al [25]. suggest specific skills needed to navigate the UME to GME transition. These include self-reflection/ self-awareness, organization, and the cultivation of social connections. Our participants employed each of these skills: self-awareness about adapting goals to their environment, organized methods of cataloging knowledge gaps for future review, and identifying trusted learning sources. Program leaders can benefit by teaching their learners to do the same.

Teaching was profoundly beneficial for MALs and closely resembled the pedagogical concept of Peer Assisted Learning, [46] or “'people from similar social groupings who are not professional teachers helping each other to learn and learning themselves by teaching.” Our findings align with prior work showing that not only does the act of teaching provide deep learning, but simply *preparing* to teach is more effective than traditional learning [47]. This finding makes sense as the constituent tasks of teaching sit atop Bloom’s Revised Taxonomy [48]: the ability to *analyze* pertinent material, to *evaluate* one’s understanding and ability to teach it, and to *create* and communicate understandable content. Program leaders should focus on building a culture of teaching (e.g. maximizing teaching roles, incorporating teaching skills training), and in so doing, provide additional benefit in the form of an improved LE for all [9].

### Create opportunities for collaborative learning

MALs cultivate meaningful social interactions in the LE to promote their learning. Bransen et. al. [49] observed co-regulated learning among medical students and found they shift their reliance from peers to seniors as they progress through learning. We found that our learners used both vertical (i.e. seniors and near-peers) and horizontal (peers) relationships in generating peer learning networks and trusted information sources. This observation may explain why, despite being thought integral to MAL development [6], none of our participants discussed engaging with formal programmatic coaching. Our learners created their own ad hoc coaching system, stitching together meaningful social sources of knowledge and formative feedback. It is unclear whether this is a consequence of necessity (GME coaching programs were uncommon at participating sites at the time of this study) or time in training (most of our learners were more senior learners). Furthermore, we did not specifically ask participants about the value of coaching. Future work targeting the timing and value of coaching (i.e. would junior learners benefit more than seniors from a coaching program?) would be valuable.

We recommend that program leaders facilitate learner collaborations. For our participants, peer learning networks both facilitated learning and normalized negative emotions experienced during learning. Training programs could harness these benefits by providing residents the encouragement, time, and space to engage in these experiences. In addition, the hierarchy of medicine—a significant component of the hidden curriculum—may inhibit the development of empowerment in vertical relationships, which are essential to learners as they discover and place value on trusted sources for their learning [50]. Any considerations that can level this hierarchy, such as longitudinal coaching programs [51], should be investigated.

### Relationship to the existing MAL model

The MAL conceptual model postulates four important learner characteristics – curiosity, motivation, mindset, and resilience – that figuratively power the learning process. The model also identifies two external factors, coaching and the learning environment, that enable MALs to thrive [6]. While our work focused on the LE, it provides supportive evidence for curiosity, motivation, and mindset. The impact of resilience and coaching, however, are less clear. Cutrer et. al. suggest that resilience is essential to a learner when managing stress and other challenges associated with learning, but we found that a learner’s *regulation* of their emotions plays a more dominant role. Our work also showed that specific peers and supervisors in the learning environment serve as trusted individuals who enable opportunities to teach, learn, and validate experiences and emotions. However, in contrast to the MAL model, ‘coaching’ was not discussed by our participants at any time.

### Limitations

Our study has limitations common to qualitative research, most notably that by relying on program director’s referral, we may have recruited participants who did not necessarily function as a MAL. In addition, our findings may reflect the experiential bias of our investigators, all of whom are emergency physicians.

The data presented in this study comes from an investigation originally focusing on the *Planning* stage of the MAL framework [10]. In response to our global questions meant to frame our discussion, participants provided detailed descriptions of their interaction with the LE. We acknowledge that the original focus of the work may bias interpretations.

Lastly, our study took place only at large tertiary-care academic medical centers in the United States. This may limit the generalizability of our findings to other settings.

## Conclusions

We describe how Master Adaptive Learners experience the process of learning during residency training and recommend methods to redesign curricula to optimize the LE for trainee success. By directly teaching these learning skills and strategies, we believe programs can transcend the negative effects of the hidden curriculum and create trainees capable of successfully navigating the learning environment of residency (and beyond). Further research is required to demonstrate the efficacy of our recommended interventions.

## Supplementary Information

**Additional file 1.** Focus Group Script.

## Data Availability

The datasets generated and/or analysed during the current study are not publicly available due as participants of this study did not agree for their data to be publically available, but are available from the corresponding author on reasonable request.
