# Supplementary material for: Creating a better learning environment: a qualitative study uncovering the experiences of Master Adaptive Learners in residency
Source: BMC Med Educ. 2022 Mar 4;22:141. doi: 10.1186/s12909-022-03200-5 (PMC8895544; doi:10.1186/s12909-022-03200-5)
Supplement: Supplementary file 1 — Additional file 1. Focus Group Script. [file 12909_2022_3200_MOESM1_ESM.docx]

**Supplemental Material: Focus Group Script**

Capture initial thoughts:

1. When you think about learning “how to learn” what is the first thing that comes to mind?

2. Have you participated in any formal training that taught you “how to learn”?

Key Questions:

1. Let’s discuss your current learning strategies.

Probe based on responses- if relevant, consider asking the following

• How did you develop that practice or skill? (Formal coursework, mentorship, self-taught)

• Why do you think the way you learn is effective?

• Has this changed as you have matured as a learner?

2. How do you identify a gap in your knowledge or skill?

Probe based on responses- if relevant, consider asking the following

• How did you develop that practice or skill? (Formal coursework, mentorship, self-taught)

• Why do you think this is effective for you?

• Has this process changed as you have matured as a learner?

• Do you feel that you mainly identify your own gaps in learning? Or do other people (your program leadership, faculty, etc.) identify them for you?

3. If you identify a gap, why do you prioritize certain gaps and how do you address it?

Probe based on responses- if relevant, consider asking the following:

• How did you develop that practice or skill? (Formal coursework, mentorship, self-taught)

• Are there times when you do not? Why or why not?

• How do you prioritize what to address and what not to address?

• Has this process changed as you have matured as a learner?

4. Do you set goals for your own learning? If so, how to you plan to meet your goals?

Probe based on responses- if relevant, consider asking the following:

• How did you develop that practice or skill? (Formal coursework, mentorship, self-taught)

• Has this process changed as you have matured as a learner?

5. How do you identify quality resources to use for your learning?

Probe based on responses- if relevant, consider asking the following:

• How did you develop that practice or skill? (Formal coursework, mentorship, self-taught)

• Has this process changed as you have matured as a learner?
